# Xenobiotic Effects on Intestinal Stem Cell Proliferation in Adult Honey Bee (*Apis mellifera* L) Workers

**DOI:** 10.1371/journal.pone.0091180

**Published:** 2014-03-07

**Authors:** Cordelia Forkpah, Luke R. Dixon, Susan E. Fahrbach, Olav Rueppell

**Affiliations:** 1 Department of Biology, University of North Carolina, Greensboro, North Carolina, United States of America; 2 Department of Biology, Wake Forest University, Winston-Salem, North Carolina, United States of America; French National Institute for Agricultural Research (INRA), France

## Abstract

The causes of the current global decline in honey bee health are unknown. One major group of hypotheses invokes the pesticides and other xenobiotics to which this important pollinator species is often exposed. Most studies have focused on mortality or behavioral deficiencies in exposed honey bees while neglecting other biological functions and target organs. The midgut epithelium of honey bees presents an important interface between the insect and its environment. It is maintained by proliferation of intestinal stem cells throughout the adult life of honey bees. We used caged honey bees to test multiple xenobiotics for effects on the replicative activity of the intestinal stem cells under laboratory conditions. Most of the tested compounds did not alter the replicative activity of intestinal stem cells. However, colchicine, methoxyfenozide, tetracycline, and a combination of coumaphos and tau-fluvalinate significantly affected proliferation rate. All substances except methoxyfenozide decreased proliferation rate. Thus, the results indicate that some xenobiotics frequently used in apiculture and known to accumulate in honey bee hives may have hitherto unknown physiological effects. The nutritional status and the susceptibility to pathogens of honey bees could be compromised by the impacts of xenobiotics on the maintenance of the midgut epithelium. This study contributes to a growing body of evidence that more comprehensive testing of xenobiotics may be required before novel or existing compounds can be considered safe for honey bees and other non-target species.

## Introduction

The western honey bee, *Apis mellifera* (L), is the most important managed pollinator worldwide and provides economically important pollination services in natural and agricultural ecosystems [1], [2]. Despite their significance to agriculture, the number of managed honey bee colonies in the United States has declined over the past decades [3]. Since 2006, severe annual losses have been reported by beekeepers in conjunction with declining honey bee health and a syndrome of collapsing colonies that accounts for some of these losses [4], [5]. This colony collapse syndrome is characterized by the rapid disappearance of adult worker honey bees, arguing for research on adult honey bee health.

The causes of the observed decline in honey bee health are poorly understood [5], [6]. Presumably, these causes are complex and heterogeneous with multiple, potentially interacting contributors [7], [8], [9]. Novel pathogens such as Israeli acute paralysis virus and combinations of parasites and pathogens have been associated with declining honey bee health in laboratory studies [10] and large-scale surveys [11], [12]. General management stress reflecting changes in beekeeping practices and inadequate nutrition may also play important roles [13],[14]. Additionally, pesticides and other xenobiotics have been associated with mass killings of honey bees [15], and novel compounds, formulations, and applications may contribute to recent declines in honey bee health [16], [17], [18].

Honey bees are exposed to a large number of xenobiotics, some of which accumulate in their hives [16], [18]. Over 120 pesticides and metabolites have been identified to enter the hive with returning foragers or as a result of direct application by beekeepers [19], [20]. This large number is concerning because substances can harm honey bee health via synergistic interactions [16], [21], [22]. Modern systemic insecticides are incorporated into all plant parts, including the pollen and nectar that honey bees collect [23]. Through food-storage and -sharing these substances are distributed throughout the hive although substances that are directly applied to the hive, such as the miticides coumaphos and fluvalinate, are typically found in higher concentrations [18], [21], [24].

Field-relevant concentrations of some pesticides not only kill honey bees but also produce sublethal effects detectable as behavioral deficiencies [25], [26], [27], [28], shortened lifespan [29], [30], or increased susceptibility to diseases [8], [31], [32]. Because many pesticides target the nervous system, tests of sublethal effects on honey bees have concentrated primarily on behavior and direct measures of neuronal activities [33], [34]. Sublethal effects on other functions and organs have been rarely studied, although pesticides and other xenobiotics are known to affect several physiological functions. For example, compromised hypopharyngeal gland development caused by exposure of nurse bees to four different pesticides [35] can be linked to decreased brood production at the colony level [36]. Exposure of the midgut epithelium of honey bee larvae to sublethal concentrations of a broad range of pesticides resulted in increased apoptosis [37]. Both of these observations predict smaller colony sizes that eventually translate into reduced colony survival [38]. Neither of these studies, however, directly addresses the topic of sublethal physiological effects in adult workers. This gap in the literature is significant given a context of colony collapse without reduced brood production [9].

The digestive system is a critical organ for honey bee health because it is the site of contact with many pathogens and xenobiotics [39], [40]. The midgut epithelium is for many pathogens the principal barrier to invasion of the honey bee host, and it is the main site for establishment of other pathogens, such as *Nosema* sp. [41]. Additionally, the midgut epithelium is responsible for detoxification of ingested xenobiotics [42], and some insecticides specifically target the midgut epithelium [40], [43]. Damage to the midgut epithelium of honey bees has also been reported as a consequence of acute exposure to the insecticides malathion, deltamethrin, and thiamethoxam [44]. This spatial overlap between immunity and detoxification may facilitate synergistic interactions between pesticides and pathogens to the detriment of honey bee health [7], [39].

**Figure 1:**
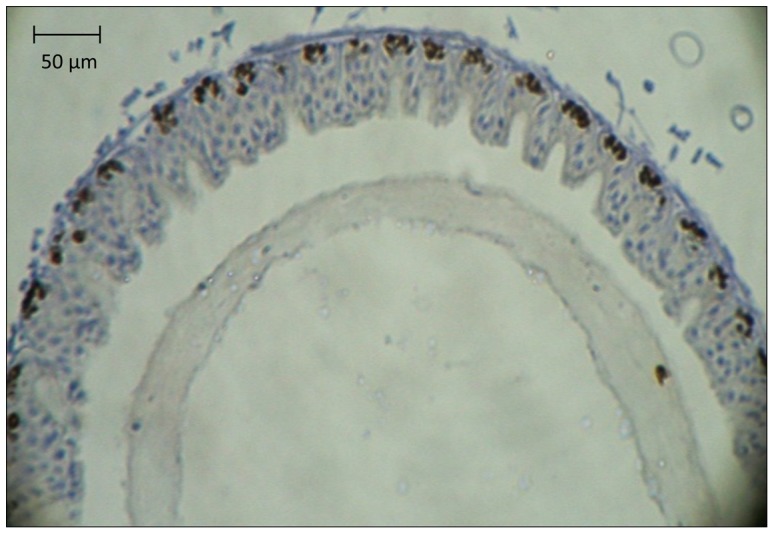
Cross section of the honey bee midgut, showing the midgut epithelium consisting of discrete crypts. The peritrophic membrane is visible in the midgut lumen. In the midgut epithelium, BrdU-labeled nuclei are brown, indicating that DNA replication occurred during the 24 h exposure to the marker. An index of proliferative activity has been developed based on counting the number of labeled nuclei in 10 µm thick cross sections relative to the number of active crypts. This index can be used to rank proliferative activity in different samples and assess possible sublethal effects of ingested xenobiotics on the midgut epithelium. Sections are counterstained with hematoxylin (in blue) to facilitate detection of crypts and other tissue features.

The midgut epithelium is the only tissue of adult honey bees that exhibits widespread cell proliferation [45]. Proliferation also occurs in the midgut of stingless adult bees, although at a lower rate than reported for honey bees. [46]. Proliferative cells (Figure 1) continuously replace the columnar and goblet cells that form the functional epithelium [47], [48], [49]. The proliferative activity of the intestinal stem cells (ISCs) varies with age and social function and responds dynamically to high digestive activity [45], [50]. The proliferation rate of the ISCs could therefore be a sensitive indicator of sublethal effects of ingested xenobiotics in the honey bee. On the one hand, toxic effects may increase the rate of proliferation by increasing the demand for cellular replacement. If replicative capacity of the ISCs is unable to compensate, epithelial function may be compromised and lifespan may be shortened. On the other hand, toxins may directly damage the ISCs, directly resulting in a decreased proliferation rate which may also compromise epithelial function and shorten lifespan.

We have examined the impact of a number of pesticides and other xenobiotics on ISC proliferation in honey bees. To investigate this potential mode of xenobiotic action, we used relatively high doses in a controlled cage environment. We concomitantly monitored survival but our focus was on the question of whether ISC proliferation is altered by sublethal exposure to common xenobiotics.

## Materials and Methods

### Experiment 1

Ten xenobiotics were studied along with solvent controls (Table 1). We used colchicine, an inhibitor of mitosis [51], as a control to demonstrate that our method was sensitive enough to detect the inhibition of ISC proliferation by a xenobiotic [45]. The insect steroid 20-hydroxyecdysone was selected as a positive control because of previous reports of a positive effect of this hormone on ISC proliferation in other insect species [52]. The trials involved monitoring survival during continuous exposure to one concentration of each xenobiotic over seven days, followed by a standardized assessment of intestinal stem cell proliferation. The chosen concentrations either represented the maximum concentrations reported from bee hives in the literature or, in the case of compounds typically applied to colonies by beekeepers, the maximum allowable dose per manufacturer instructions.

**Table 1:** Xenobiotics tested in the first experiment for effects on the intestinal stem cell proliferation.

Xenobiotic	Supplier	Dosage	Source for selecting concentrations
Fumagillin	Mann Lake Ltd	2 mg/g	Highest dose allowed per manufacturer guidelines
Tau-fluvalinate*	Mann Lake Ltd	Permanent exposure	Practical dosage under experimental conditions exceeds manufacturer guidelines
Tetracycline	Sigma- Aldrich	3 mg/g	[60]
Imidacloprid	Sigma-Aldrich	500 ppb	[16]
Coumaphos	Sigma-Aldrich	5000 ppb	[16]
Chlorothalonil	Fluka	1000 ppb	[16]
Methoxyfenozide	ChemSevice Inc.	400 ppb	[16]
Colchicine	Sigma-Aldrich	5 mg/g	[51]
20-Hydroxyecdysone	MP Chemicals	200 ppb	[68]
DMSO	Acros Chemical	0.01 mg/g	Control for imidacloprid
Acetone	Mallinckrodt Chemicals	0.1 mg/g	Control for methoxyfenozide, coumaphos, and chlorothalonil
Isopropanol	Fisher	200 ppb	Control for 20-hydroxyecdysone
Water	N/A	N/A	Control for tetracycline, colchicine, tau-fluvalinate, and fumagilin

* Tau-fluvalinate was exposed to bees using the commercial Apistan strip. A half of one standard strip was placed in each cage for the indicated exposure time per day for 7 days.

Workers (*Apis mellifera* L) from 4–10 hives maintained at the University of North Carolina at Greensboro bee yard were used. Colonies were maintained following standard practices without chemical disease control or artificial diets. Combs with ready-to-emerge workers were transferred to an incubator (complete dark cycle, 35°C, 60% rel. hum.) and collected from the combs upon emergence. Newly emerged bees were randomly assigned to treatment or control groups. Four groups of 25 bees per treatment were kept in separate Plexiglas feeding cages (10 cm×7.5 cm×10 cm) in an incubator (complete dark cycle, 33°C, 60% rel. hum.), fed *ad libitum* queen candy (9∶3∶1, powdered sugar: water: honey), and provided with water. Dead bees were removed and counted from the cages daily. Although cage studies are widely used in honey bee research [53], they can be problematic [54] and have been reported to compromise the natural colonization of the gut by bacteria [55]. We preferred the controlled cage environment for these initial studies because our goal was to link a known xenobiotic exposure to quantitative effects on ISC proliferation.

All substances except tau-fluvalinate were mixed with the queen candy food for direct delivery to the midgut epithelium. Tau-fluvalinate was delivered via Apistan strips (Zoëcon, USA), the form typically used by beekeepers. Two of the four tau-fluvalinate cages were terminated after three days instead of the planned seven day exposure to ensure that a sufficient number of living honey bees could be obtained for our studies of ISC proliferation. For all other treatments, living honey bees were collected from the cages after seven days for ISC proliferation assays.

### Experiment 2

On the basis of the results of the first experiment only methoxyfenozide, tetracycline, and tau-fluvalinate were tested further in large scale studies, using three different dosages (Table 2). Because of the potential for synergistic effects [22], a combination of tau-fluvalinate and coumaphos was also tested. The experiment comprised eleven trials, each with its own water or acetone control groups to account for seasonal effects and the use of honey bees from different sources. As described, newly emerged bees were caged, housed in an incubator, and fed xenobiotics in food provided *ad libitum* for seven days. In this study, food was provided as a 30% sucrose solution in liquid feeders. Two-four replicate cages were used per treatment, with 120–155 bees housed per cage (same dimensions as in Experiment 1). Survival was monitored daily, and a subset of the surviving bees assayed for ISC proliferation after 7 days (see below). Fresh and dry weights of the head and thorax of random samples of additional bees from all treatments were determined to test for differences in food uptake.

**Table 2:** Summary of the second experiment testing different dosages of select xenobiotics for effects on intestinal stem cell proliferation.

Xenobiotic	Supplier	Dosage		
		Low	Mid	High
Methoxyfenozide	ChemSevice Inc.	40 ppb	400 ppb	2000 ppb
Tau-fluvalinate*	Mann Lake Ltd	3 minutes randomized	3 minutes sequential	15 minutes
Tetracycline	Sigma-Aldrich	1.2 µg/g	30 µg/g	60 µg/g
Tau-fluvalinate* and 500 ppb Coumaphos	Mann Lake Ltd Sigma-Aldrich	3 minutes randomized	3 minutes sequential	15 minutes

* Tau-fluvalinate was exposed to bees using the commercial Apistan strip. A full strip was placed in each cage for the indicated exposure time per day for 7 days.

After the collection on day 7, the remaining honey bees were continued to be daily monitored for survival in their cages without xenobiotic exposure: They were provisioned with distilled water and sucrose solution. A second sample of honey bees of each treatment group was assayed for delayed treatment effects on ISC proliferation between ages 19–22 days, or earlier if mortality of the experimental cohort exceeded 90% before that age.

### ISC Proliferation Assay

**Figure 2:**
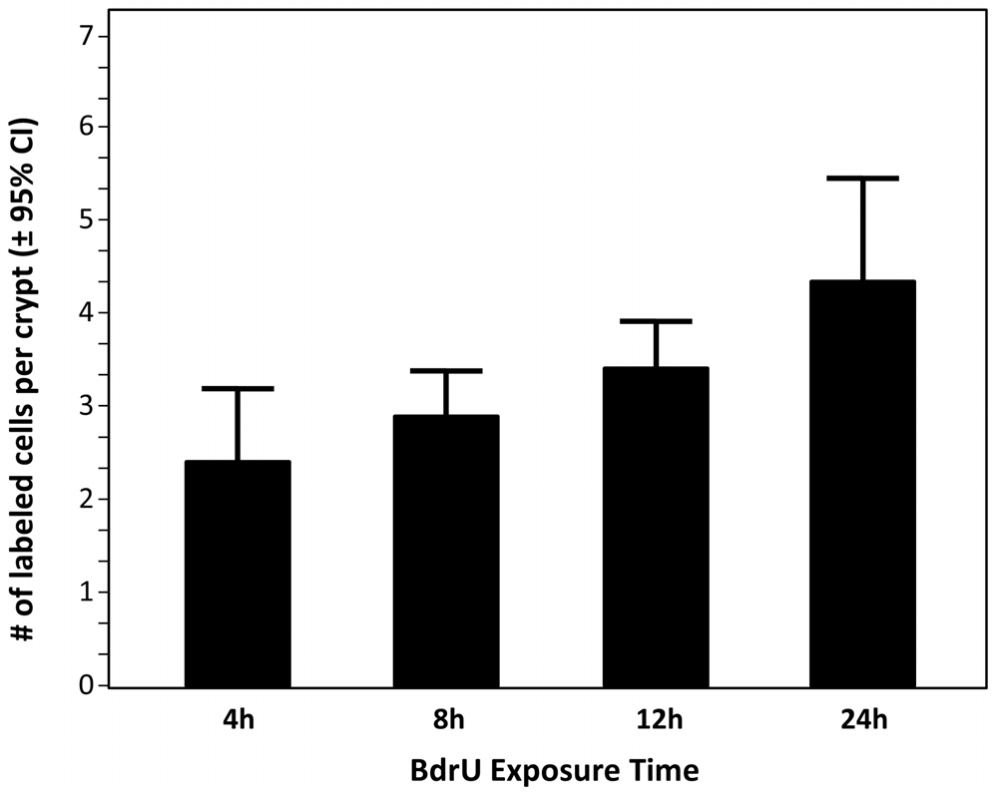
The number of labeled nuclei in a single midgut cross-section is a function of duration of exposure to BrdU. This presumably reflects the number of cell cycle events that occur during the exposure. To control for this effect, a standardized 24-individual variation among samples in this experiment was lower after 8 and 12 h.

Following our previous methods [45], [50], assessment of proliferation rate of intestinal stem cells relied on immunohistochemical labeling of the thymidine analog 5-bromo-2-deoxyuridine (BrdU) incorporated into newly synthesized DNA. Briefly, workers without signs of morbidity such as reduced mobility or responsiveness to stimuli were selected for this assay. These individuals were fed 5 mg/ml BrdU (Life Technologies, CA) in queen candy *ad libitum* for a 24-hour period. Shorter feeding periods were evaluated in a pilot study with newly emerged workers (Figure 2), and a 24-hour period was selected for the actual experiments because this survival reliably produced a substantial number of labeled nuclei.

Only individuals that appeared healthy after this feeding period were selected for analysis. Dissected midguts rinsed with saline were fixed in Carnoy's fixative for 24 hours and embedded in Paraplast (Thermo Fisher Scientific, MA) for sectioning (10 µm) using a HM315-Microm microtome (Thermo Fisher Scientific, MA). Sections were mounted on Superfrost Fisher plus microscope slides (Thermo Fisher Scientific, MA), dewaxed in xylene, rehydrated via a graded alcohol series, and permeabilized in phosphate-buffered saline containing 0.01% Triton X-100 detergent (PBS-T; Sigma-Aldrich, MO). Samples were denatured with 2N hydrochloric acid, washed in phosphate-buffered saline (PBS), blocked with normal goat serum (Thermo Fisher Scientific, MA), and incubated with anti-BrdU antibody (Phoenix Flow Systems, PRB1U) for 24 h at 4°C. After several washes in PBS-T and PBS, sections were incubated at room temperature for two hours with a peroxidase-conjugated anti-mouse secondary antibody (Jackson ImmunoResearch Laboratories, PA), washed again, and incubated with the chromogen diaminobenzidine (Sigma-Aldrich, MO). All nuclei containing DNA synthesized after ingestion of BrdU were labeled with a dark brown reaction product. Slides were counterstained for approximately five minutes using Gill hematoxylin (Thermo Fisher Scientific, MA) followed by 0.1% sodium-bicarbonate solution for one minute. After dehydration in ethanol, the tissue was cleared with CitriSolv (Thermo Fisher Scientific, MA). Slides were coverslipped using Permount and viewed under a Nikon Eclipse E200 microscope.

In the first experiment, all BrdU-labeled nuclei and active centers of proliferation (crypts) were counted in one randomly selected intact section per individual (Figure 1). An active crypt was defined as any containing one or more cells with a labeled nucleus. The average number of labeled nuclei per crypt visible in the selected section was calculated. In the second experiment, the labeled nuclei of 10–22 random crypts from multiple, arbitrarily selected intact cross sections were counted, and the average number of labeled nuclei per crypt was determined to reduce bias associated with analysis of a single section. Observers evaluated slides without knowledge of treatment group identity.

### Analyses

In the initial screening experiment, differences in survival between xenobiotic exposed-groups and control groups were assessed by simple contingency analyses with Yates' correction because standard survival estimates and statistical comparisons could not be computed in groups with 100% survival until the end of the experiment. In the follow-up experiments, survival was compared among the treatment and vehicle control groups by pairwise Kaplan-Meier analysis (log-rank tests), censoring any individuals that were sampled for quantification of their intestinal stem cell proliferation or weight determination. We separately assessed acute mortality (during xenobiotic exposure) and legacy mortality (after xenobiotic exposure was terminated). Cages were treated as separate replicates in the overall evaluation of each experimental treatment.

In the first experiment, the effects of each xenobiotic on the number of labeled nuclei, active crypts, and number of labeled nuclei per crypt were assessed by simple ANOVAs. In the second experiment, the effect of each xenobiotic on the number of labeled nuclei per crypt was analyzed by ANOVA using age group (acute versus legacy effects) as one independent fixed factor and treatment as the second factor. The treatment factor divided the samples into honey bees that were exposed to the three different concentrations of each xenobiotic and the appropriate vehicle control. The overall analyses were followed by separate analyses of the two age groups in which interactions between treatment and age were indicated. Because of unequal variances among groups, *post hoc* comparisons among the different doses of a specific xenobiotic treatment were performed with Dunnett's T3 test.

## Results

### Experiment 1

**Figure 3:**
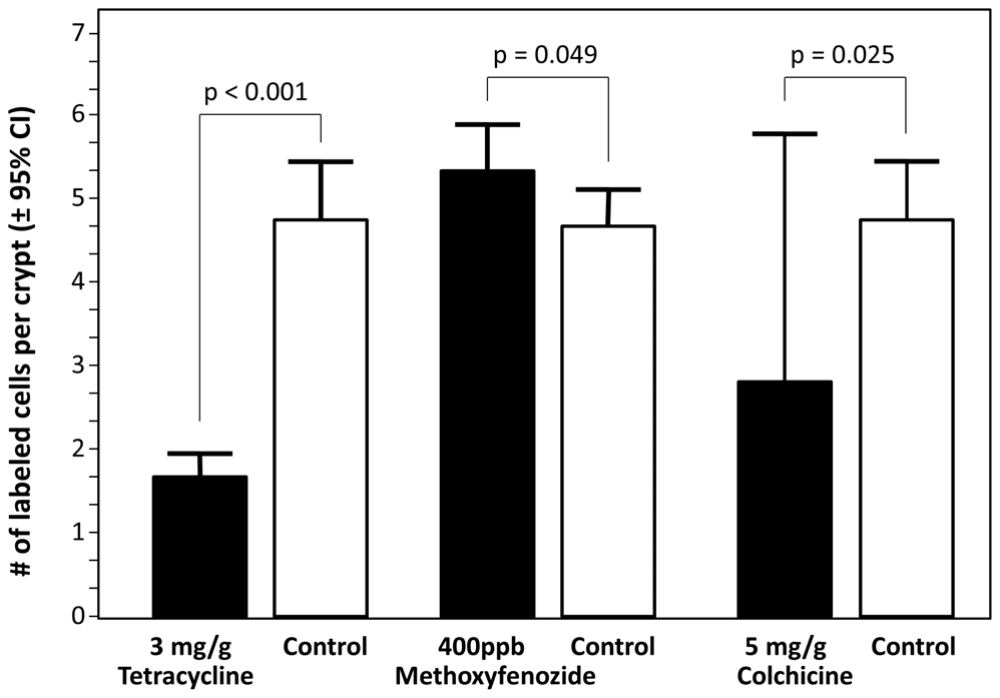
In the first experiment, 3 of the 12 xenobiotics tested had significant effects on the number of labeled nuclei per active crypt. While colchicine and tetracycline decreased the proliferation of ISCs, methoxyfenozide increased activity. Means are shown with 95% confidence intervals.

ISC proliferation was significantly affected by feeding on colchicine, tetracycline, and methoxyfenozide, but not by feeding on fumagilin, imidacloprid, coumaphos, chlorothalonil, or by fluvalinate treatment (Table 3). Compared with untreated controls, colchicine significantly reduced the number of labeled nuclei per active crypt (2.8 versus 4.7). Workers that fed on tetracycline had fewer active crypts per section (32.8 versus 55.3), fewer labeled nuclei per section (55.9 versus 254.9), and fewer labeled nuclei per active crypt (1.7 versus 4.7). In contrast, methoxyfenozide significantly increased labeled nuclei per section (324.9 versus 251.5) and per crypt (5.3 versus 4.7), relative to controls (Figure 3). The survival of individuals across experimental groups was positively correlated with the average number of labeled nuclei per crypt (Pearson's R_P_ = 0.79, n = 13, p = 0.001). The proportion of surviving individuals varied among experimental groups from 6.4% to 100%. None of the solvent controls affected honey bee survival but survival was significantly reduced by the high experimental exposure to colchicine (χ^2^ = 154.7, p<0.001), tetracycline (χ^2^ = 169.2, p<0.001), fluvalinate (χ^2^ = 119.2, p<0.001), fumagillin (χ^2^ = 76.4, p<0.001), imidacloprid (χ^2^ = 20.9, p<0.001), and coumaphos (χ^2^ = 6.4, p = 0.012) relative to their respective controls.

**Table 3:** Xenobiotic feeding effects* on ISC proliferation pooled across replicate cages.

Xenobiotic	Effect on # of labeled cells per cross-section	Effect on # of crypts per cross-section	Effect on # of labeled cells per crypt
Fumagillin	F_(1,17)_ = 0.0, p = 0.936	F_(1,17)_ = 0.0, p = 0.900	F_(1,17)_ = 0.4, p = 0.536
Tau-fluvalinate	F_(1,31)_ = 0.0, p = 0.946	F_(1,20)_ = 0.2, p = 0.626	F_(1,20)_ = 1.8, p = 0.195
**Tetracycline**	**F_(1,14)_ = 56.3, p<0.001**	**F_(1,13)_ = 17.7, p = 0.001**	**F_(1,13)_ = 56.3, p<0.001**
Imidacloprid	F_(1,16)_ = 0.0, p = 0.983	F_(1,16)_ = 0.0, p = 0.871	F_(1,16)_ = 0.0, p = 0.967
Coumaphos	F_(1,19)_ = 0.6, p = 0.440	F_(1,17)_ = 0.2, p = 0.678	F_(1,17)_ = 3.2, p = 0.091
Chlorothalonil	F_(1,23)_ = 0.4, p = 0.544	F_(1,20)_ = 0.1, p = 0.725	F_(1,20)_ = 0.8, p = 0.384
**Methoxyfenozide**	**F_(1,21)_ = 6.9, p = 0.016**	F_(1,20)_ = 3.4, p = 0.079	**F_(1,20)_ = 4.4, p = 0.049**
**Colchicine**	F_(1,11)_ = 4.2, p = 0.065	F_(1,9)_ = 0.4, p = 0.534	**F_(1,10)_ = 7.2, p = 0.025**
20-Hydroxyecdysone	F_(1,20)_ = 0.3, p = 0.592	F_(1,18)_ = 1.3, p = 0.273	F_(1,18)_ = 0.3, p = 0.596

^*^Significant effects in bold.

### Experiment 2

The experimental groups did not differ significantly in fresh or dry weights of the head (F^fresh^_(9,379)_ = 1.4, p = 0.167, F^dry^_(9,379)_ = 1.2, p = 0.267) or dry weight of the thorax (F^dry^_(9,379)_ = 1.3, p = 0.218). In contrast, thorax fresh weight was significantly affected (F^fresh^_(9,379)_ = 2.5, p = 0.010). *Post hoc* comparisons revealed that a significantly lower thorax weight was found in the acetone control group than in the water control and in the highest tetracycline dosage group.

**Figure 4:**
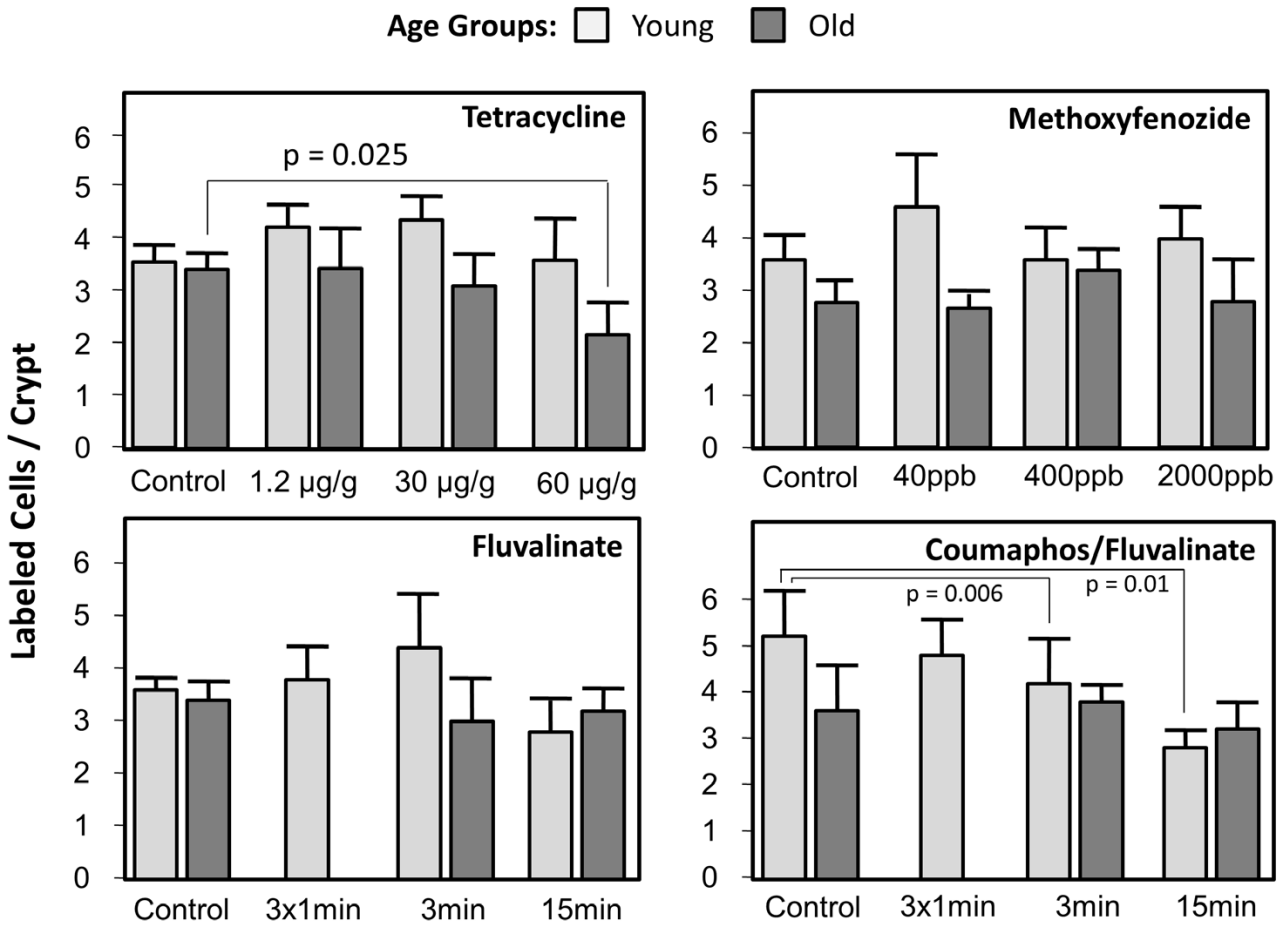
ISC proliferation, indicated by the number of BrdU-labeled nuclei per active crypt, was significantly decreased in older bees. The combination of coumaphos and fluvalinate reduced proliferation measured immediately after treatment, while tetracycline decreased proliferation only at older ages, over ten days after exposure to the xenobiotic had ended.

Xenobiotic feeding effects on the number of labeled nuclei per active crypt were variable (Figure 4). Tetracycline showed significant concentration (F_(3,99)_ = 2.8, p = 0.042), age group (F_(1,99)_ = 18.5, p<0.001), and interaction (F_(3,99)_ = 2.9, p = 0.040) effects. Overall, sections contained more labeled nuclei directly after termination of treatment than two weeks later. Differences among treatments were not significant in the young age group directly after xenobiotic exposure (F_(3,57)_ = 2.4, p = 0.081), but the highest dosage of tetracycline was associated with a significant decline in the number of labeled nuclei per crypt compared with the control group in the older age group (F_(3,42)_ = 3.5, p = 0.025). Analysis of the results of the methoxyfenozide experiment revealed a smaller number of labeled nuclei in older bees (F_(1,71)_ = 29.0, p<0.001), no overall effect of treatment (F_(3,71)_ = 1.1, p = 0.349), but a significant interaction between the two factors (F_(3,71)_ = 3.5, p = 0.019). Analyzed separately, no significant effect of treatment was apparent in either age group (young: F_(3,34)_ = 2.5, p = 0.074; old: F_(3,37)_ = 1.4, p = 0.250). Fluvalinate alone exhibited no overall age group (F_(1,98)_ = 2.3, p = 0.129) or treatment (F_(3,98)_ = 1.1, p = 0.371) effects but a significant interaction effect (F_(2,98)_ = 3.1, p = 0.048). Separate analyses did not reveal specific treatment effects in either age group (young: F_(3,57)_ = 2.3, p = 0.087; old: F_(2,41)_ = 0.5, p = 0.616). Coumaphos and fluvalinate in combination showed a significant treatment effect (F_(3,58)_ = 6.8, p = 0.001), no age group effect (F_(1,58)_ = 3.1, p = 0.084), and a significant interaction between the two factors (F_(2,58)_ = 4.0, p = 0.023). Treatment significantly affected the labeled nuclei per active crypt in the younger group (F_(3,30)_ = 7.8, p = 0.001), with the highest dosage significantly reducing the counts relative to the control and lowest dosage. In the older group, no significant treatment effect was found (F_(2,28)_ = 1.0, p = 0.385).

Across all treatment groups, there was no significant relation between ISC proliferation directly after xenobiotic exposure and its measure at older ages (Rs = 0.02, n = 13, p = 0.943). The average ISC proliferation in the groups sampled at the older age was positively associated with survival after treatment (R_S_ = 0.67, n = 13, p = 0.013), while no association between ISC proliferation and survival at the younger age (during xenobiotic exposure) was found (R_S_ = −0.19, n = 15, p = 0.499).

**Figure 5:**
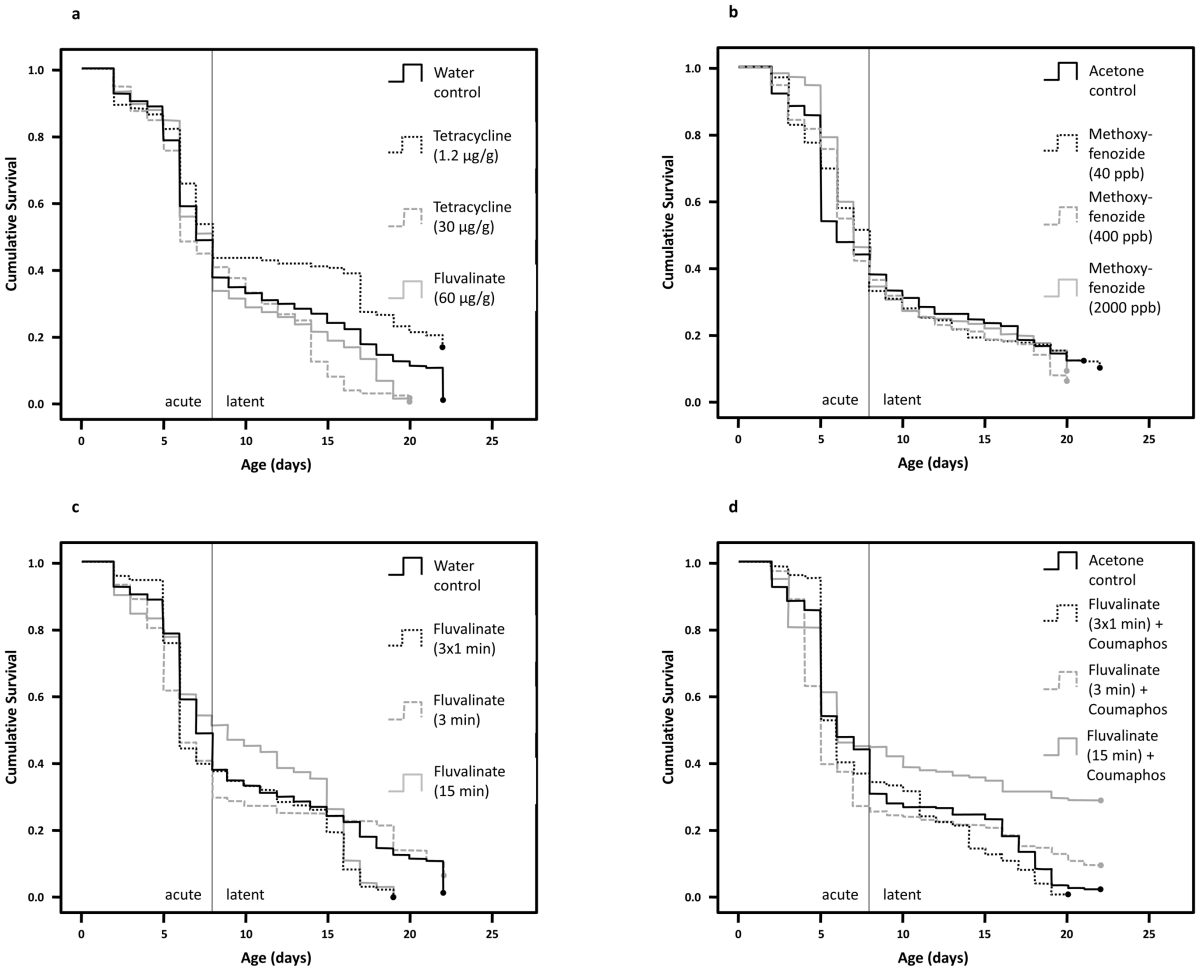
Survival of worker cohorts under high-density cage conditions was lower than in the initial screening experiment and varied inconsistently among treatments. Different panels summarize cumulative survival of honey bee workers grouped from different cage replicates according to the tested xenobiotic: (a) tetracycline, (b) methoxyfenozide, (c) tau-fluvalinate, and (d) the combination of tau-fluvalinate and coumaphos. Acute mortality effects were measured during the first 7 days of exposure, while legacy mortality effects were measured on the days after treatment had ended.

Mortality in the caged experimental cohorts was generally higher than in the first experiment with seven-day survival ranging from 44–68% and significant variation among cages of the same treatment groups, including control groups (see File S1). Overall, the acute mortality was different among treatment groups for tetracycline (χ^2^ = 10.1, p = 0.018; Figure 5a), methoxyfenozide (χ^2^ = 8.8, p = 0.032; Figure 5b), fluvalinate (χ^2^ = 18.7, p<0.001; Figure 5c), and the combination of fluvalinate and coumaphos (χ^2^ = 38.9, p<0.001, Figure 5d). After Bonferroni correction, only the 3-minute fluvalinate treatment (χ^2^ = 11.8, p_corr_ = 0.002) and the 3-minute fluvalinate exposure combined with coumaphos (χ^2^ = 31.0, p_corr_<0.001) increased mortality compared with the respective solvent controls.

Overall legacy mortality after the treatment was different among experimental groups for tetracycline (χ^2^ = 113.6, p<0.001; Figure 5a), fluvalinate (χ^2^ = 27.3, p<0.001; Figure 5c), and the combination of fluvalinate and coumaphos (χ^2^ = 114.4, p<0.001, Figure 5d). Treatments that significantly increased legacy mortality relative to their respective controls were the medium (χ^2^ = 32.5, p_corr_<0.001) and high (χ^2^ = 33.7, p_corr_<0.001) dose of tetracycline and the 3×1 min exposure of fluvalinate (χ^2^ = 11.5, p_corr_ = 0.002).

## Discussion

This study demonstrated that select xenobiotics can decrease the proliferative rate of ISCs of adult worker honey bees. Reduced ISC proliferation represents a novel, possibly important effect of xenobiotics because the midgut epithelium provides the first line of defense against many pathogens, is responsible for nutrient uptake, and detoxifies many ingested toxins [39].

Most of the tested substances did not significantly affect ISC proliferation although they were directly ingested and therefore must have come into close contact with the midgut epithelium of the studied honey bees. This finding contrasts with widespread pesticide effects on apoptosis in the midgut of honey bee larvae [37], suggesting that juvenile stages might be more susceptible to pesticides than adults. Only colchicine (included as a technical control), tetracycline, methoxyfenozide, and a combination of fluvalinate and coumaphos showed effects on ISC proliferation. The effects were moderate, dose-dependent, and inconsistent between experiments for methoxyfenozide. Our overall results indicate that the replication rate of ISCs is quite robust after ingestion of most xenobiotics under cage conditions. In contrast, in-hive studies have shown that ISC replication rates decrease in worker honey bees with age and reduced digestive activity [45], [50]. Future experiments that better mimic hive conditions and field-relevant exposure levels will be necessary to assess the threat of xenobiotics for intestinal health of honey bees.

Xenobiotic-induced alteration of ISC proliferation may directly harm the affected worker honey bees, causing an increase in immediate or delayed mortality. Overall, our results suggest that reduced ISC proliferation is associated with mortality. Specifically in the second experiment, one of the coumaphos and fluvalinate combination treatments decreased ISC proliferation and survival during exposure; the high tetracycline dosage exhibited delayed effects on both ISC proliferation and mortality. Under field conditions, these effects would result in smaller and/or collapsed colonies due to increased mortality of adult workers. However, we cannot rule out that xenobiotic-induced alteration of ISC proliferation also occurs independently of increased mortality. Under field conditions such effects may increase individual disease susceptibility, for example to *Nosema* [7], [56], and compromise the physiological capacity of nurse bees to produce sufficient brood food. Additional studies are needed to address these questions because exposures to sublethal levels of xenobiotics are likely to be more common than exposures to lethal levels [32], [38] and sublethal effects are important but difficult to integrate into pesticide regulation [33], [57].

In the first experiment, mortality in the cages was increased by several xenobiotics presented at high dosages. Therefore, we employed lower dosages in the second experiment and extended our mortality and ISC proliferation measurements to include potentially subtle long term effects on ISC proliferation. The sample sizes required for the additional long term analyses resulted in crowded cages and higher mortality, even in the untreated control cages. The increased mortality likely reflects a variety of factors, including poorer hygiene and competition for access to the feeder [53]. However, the determination of worker body weight at the end of the second experiment did not indicate significant differences in food intake between xenobiotic and control groups. We excluded all moribund individuals when assessing ISC proliferation but the concomitant assessment of potential mortality effects of the administered treatment is problematic, particularly because significant variation among replicate cages existed and effects on mortality were inconsistent when cages were analyzed separately (see File S1). Thus, we are reluctant to label any of the measured effects as lethal or sublethal, although mortality was increased by treatments that reduced ISC proliferation. Similarly, the insecticides thiamethoxam, deltamethrin, and malathion have been shown to disrupt the integrity of the honey bee midgut at concentrations that increase mortality [44].

Tetracycline is widely used by beekeepers to combat *Paenibacillus larvae* and *Melissococcus plutonius*, the bacterial agents of American and European foulbrood, respectively [58], but it is a general antibiotic with a wide range of target microorganisms [59]. Compared with controls, caged honey bees exposed to tetracycline exhibited lower ISC proliferation in both experiments. In the first experiment, a dosage that was 1000-fold higher than that typically found in hives [60] significantly reduced ISC proliferation directly after the seven days of treatment. In the second experiment a 50-fold reduced dosage, but not a 100-fold or 2500-fold reduced dosage, also reduced ISC proliferation in the long term. No short-term effects were observed for the lower dosages in the second experiment. Thus, exposure of honey bees to very high doses of tetracycline may result in acute deterioration of the gut physiology or compromise ISCs directly, while lower concentrations of tetracycline appear to produce a delayed effect. The delayed effect could be due to changes in the intestinal microbial community that can disrupt honey bee health [61], [62]. Although we did not monitor the intestinal microbiome, our results could be explained by an interaction between the intestinal microbiome and the physiology of its honey bee host, similar to findings reported in Drosophila that linked the intestinal microbiome to stem cell proliferation [63].

The insect growth regulator methoxyfenozide has not been demonstrated to be harmful to adult honey bees [64], but this compound also accumulates in honey bee hives at significant concentrations [18]. The results of our first experiment suggested that methoxyfenozide may have physiological effects in honey bees by stimulating ISC proliferation. This observation is consistent with the role of methoxyfenozide as an ecdysteroid agonist in the insect midgut [52], [65]. In contrast, direct feeding of 20-hydroxyecdysone, did not affect ISC proliferation, which may reflect the efficient metabolic conversion of the natural hormone by the gut [66]. Under the crowded conditions of the second experiment, the increases in acute ISC proliferation produced by methoxyfenozide exposure were not significant, and at the older age the low exposure group actually showed a slightly lower number of labeled nuclei than the respective control bees. Thus, the effect of methoxyfenozide on the ISCs is subtle and might not have any health consequences, particularly when considering that the concentrations found in honey bee hives are typically lower than the tested concentrations [18].

Fluvalinate is used by beekeepers to control *Varroa* and tracheal mites. The high dosage of fluvalinate in the first experiment proved so toxic that we quantified ISC proliferation after three days, a time at which most of the exposed workers had already died. At this time no significant effect on ISC proliferation was apparent. In the second experiment we reduced the daily exposure to the fluvalinate strip by over 80-fold, resulting in lower mortality. No effect on ISC proliferation was found after 7 days of exposure and 2 weeks after exposure was terminated. The second commonly used miticide, coumaphos, also did not show significant effects on ISC proliferation in the first experiment despite a very high dosage only found in rare cases under natural conditions [18], [30]. However, the combination of coumaphos and fluvalinate significantly decreased short-term ISC proliferation. Concomitantly, this combination treatment decreased survivorship at all dosage levels relative to the corresponding fluvalinate-only treatments (log rank tests: 3×1 min: χ^2^ = 15.0, p<0.001; 3 min: χ^2^ = 3.9, p = 0.049; 15 min: χ^2^ = 5.9, p = 0.015). Thus, the mortality data and ISC proliferation rates indicate synergism between fluvalinate and coumaphos, which has been reported in other contexts [21], [22]. The dosages used in the second experiment may be higher than average field exposure but they fall within the limits of concentrations measured in honey bee hives [18], [67] and the findings may therefore be relevant for honey bee health. Coumaphos and fluvalinate both target primarily the nervous system: Coumaphos, when converted to its metabolite coumaphos oxon, inhibits the acetylcholinesterase enzyme and fluvalinate serves as an agonist of the voltage-gated sodium channel [22]. Our results may therefore be explained by effects on the neural control of the digestive system or changes in behavior that may have indirectly decreased ISC proliferation. However, we cannot rule out other, non-neural effects. The synergism between the two miticides may be due to inhibition of the detoxification mechanism [22].

We did not find support for the hypothesis that ISCs increase proliferation to compensate for xenobiotic damage to the midgut epithelium [37]. Instead, tetracycline and the combination of fluvalinate and coumaphos decreased ISC proliferation, suggesting direct or indirect effects that decrease ISC activity. The number of labeled nuclei per active crypt also declined with age in all control and treatment groups, except for the groups with the highest exposure to fluvalinate. This finding confirms our earlier results that ISC proliferation declines with age in honey bees [45]. The age-related decline under natural conditions may reflect the fact that digestive demand is higher in young workers, which are typically nurse bees [45]. In our cage experiments, however, workers did not transition from nursing to foraging behavior. Thus, the age-related decline of ISC proliferation occurred independently of diet or behavioral changes, suggesting the possibility of intrinsic aging of the replicative capacity of ISCs.

## Supporting Information

File S1 **Significant variability in mortality among separate cages was observed within each treatment of the second experiment.** This file details the mortality results with respect to the separate cages in each treatment. Due to unexplainable variation and the focus of our study on ISC proliferation, we omitted these details from the main text.
(DOCX)
