# Supplementary material for: Xenobiotic Effects on Intestinal Stem Cell Proliferation in Adult Honey Bee (Apis mellifera L) Workers
Source: PLoS One. 2014 Mar 7;9(3):e91180. doi: 10.1371/journal.pone.0091180 (PMC3946715; doi:10.1371/journal.pone.0091180)

**Details of the Second Experiment: Separate Evaluation of Honey Bee Mortality among Cage Replicates**

Mortality

Overall, 51.6% of the 1221 water control bees in eight replicated cages survived the first seven days (Figure S1). Survival was significantly different among replicate cages (χ^2^ = 31.9, p < 0.001) with survival ranging from 43% to 61%. Tetracycline feeding did not affect acute mortality (Figure S2) in any treatment groups (χ^2^ ranging from 0.0 to 1.0, p ranging from 0.311 to 0.992), except for one replicate of the 60 ppb treatment showing lower mortality than its water control (χ^2^ = 15.7, p < 0.001). Acute survival of methoxyfenozide-fed bees (Figure S3) was increased in both replicate cages of the middle dosage (χ^2^ = 7.2, p = 0.007 and χ^2^ = 4.8, p = 0.029) and one replicate of the high dosage (χ^2^ = 5.0, p = 0.026), and not significantly different in the remaining high dose replicate (χ^2^ = 1.8, p = 0.185) and both low dose replicates (χ^2^ = 1.2, p = 0.265 and χ^2^ = 0.2, p = 0.618) relative to its acetone controls (Figure S4).

The remaining treatments also yielded variable results. In the case of fluvalinate (Figure S5), the 15 minute treatment increased acute mortality in one replicate (χ^2^ = 20.3, p < 0.001) but decreased acute mortality in the other replicate (χ^2^ = 11.6, p = 0.001). A 3 minute exposure to fluvalinate increased mortality in both replicates (χ^2^ = 6.9, p = 0.009 and χ^2^ = 5.2, p = 0.023). In contrast, three 1 minute exposures per day to fluvalinate decreased mortality in one replicate (χ^2^ = 8.6, p = 0.003) and had no effect in the other replicate (χ^2^ = 0.5, p = 0.491). Similarly, the combination of fluvalinate and coumaphos (Figure S6) increased mortality in both replicates for the 3 minute continuous exposure (χ^2^ = 11.1, p = 0.001 and χ^2^ = 45.3, p < 0.001) and in one replicate of the 15 minute exposure (χ^2^ = 11.6, p = 0.001) but failed to show an effect in the other replicate of the 15 minute exposure (χ^2^ = 2.3, p = 0.130) and both replicates of the three 1 minute exposure treatment (χ^2^ = 2.0, p = 0.161 and χ^2^ = 0.0, p = 0.915).

Long term survival after the end of treatment between the ages of 8 and 22 days was also significantly affected by replicate, as indicated by significant differences (χ^2^ = 117.9, p < 0.001) among the water control groups (Figure S1). Therefore, treatment groups were only compared with controls measured simultaneously. Tetracycline increased long term survival (Figure S2) at the low dose in both replicates (χ^2^ = 5.5, p = 0.019 and χ^2^ = 22.9, p < 0.001) and at the high dose in one replicate (χ^2^ = 8.5, p = 0.004), while no significant effect was measured in the 2^nd^ replicate of the high dose (χ^2^ = 0.8, p = 0.374) and both replicates of the intermediate dose (χ^2^ = 3.4, p = 0.065 and χ^2^ = 0.8, p = 0.386). At the high, medium, and low concentrations, methoxyfenozide increased long-term survival in one replicate (χ^2^ = 4.7, p = 0.031, χ^2^ = 14.9, p < 0.001, χ^2^ = 6.1, p = 0.013, respectively) and did not significantly affect long-term survival in the other replicate (χ^2^ = 2.5, p = 0.117, χ^2^ = 0.1, p = 0.770, χ^2^ = 0.4, p = 0.538, respectively) (Figure S3).

Fifteen minutes of fluvalinate exposure per day decreased long term survival in one replicate (χ^2^ = 48.3, p < 0.001) but increased it in the other replicate (χ^2^ = 6.2, p = 0.013). A three minute continuous exposure to fluvalinate increased survival (replicate 1: χ^2^ = 7.5, p = 0.006) or did not affect it (replicate 2: χ^2^ = 0.0, p = 0.902). Three 1 minute exposures to fluvalinate decreased long-term survival in one replicate (χ^2^ = 6.5, p = 0.011) but did not affect it in the other replicate (χ^2^ = 0.1, p = 0.724) (Figure S5). The combination of fluvalinate treatment and coumaphos feeding (Figure S6) resulted at 15 minute fluvalinate exposure in higher long term survival in one replicate (χ^2^ = 32.8, p < 0.001) but the opposite effect in the other replicate (χ^2^ = 4.9, p = 0.026). Coumaphos paired with 3 minute continuous fluvalinate exposure let to a higher (replicate 1: χ^2^ = 36.0, p < 0.001) or equal survival (replicate 2: χ^2^ = 1.3, p = 0.251). When coumaphos was paired with three 1 minute fluvalinate exposures per day, long-term survival was increased (replicate 1: χ^2^ = 14.2, p < 0.001) or decreased (replicate 2: χ^2^ = 37.7, p < 0.001).

In sum, the mortality results in the second experiment were inconclusive due to the significant variation among cage replicates in the control and treatment groups.

Figure S1: Cumulative survivorship of worker honey bees in the water control group. Censored data due to sampling after day seven are not indicated because short-term (acute) and long-term (latent) survivorship were evaluated separately.


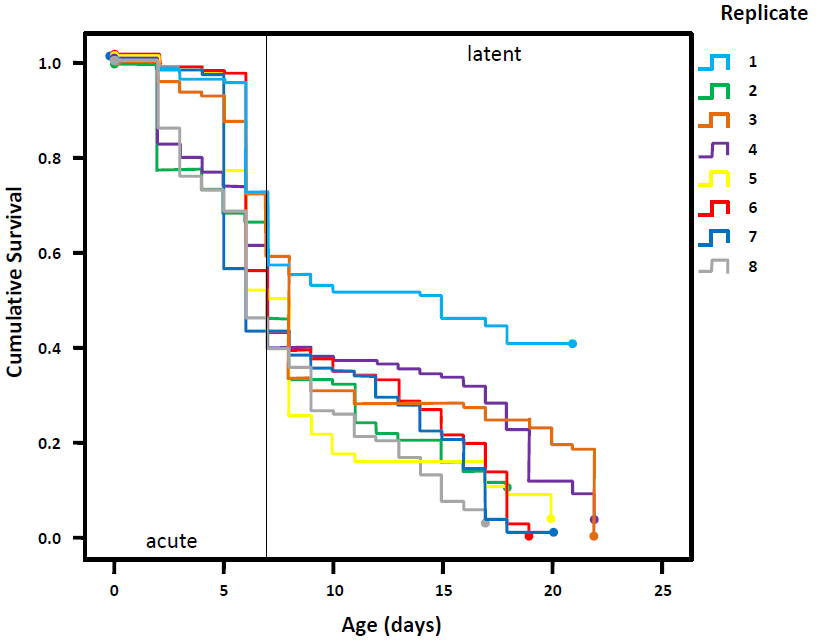


Figure S2: Cumulative survivorship of worker honey bees in the tetracycline group. Censored data due to sampling after day seven are not indicated because short-term (acute) and long-term (latent) survivorship were evaluated separately.


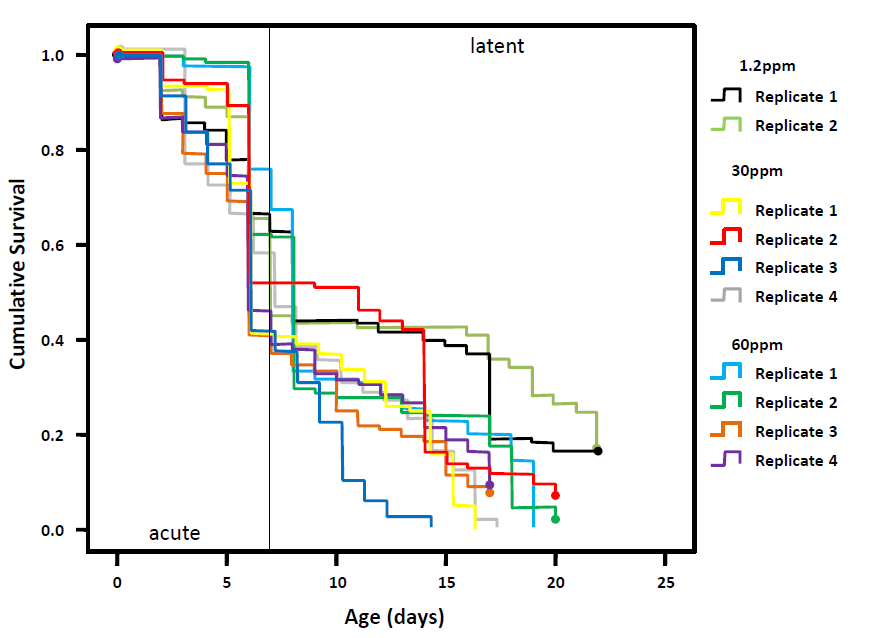


Figure S3: Cumulative survivorship of worker honey bees in the methoxyfenozide group. Censored data due to sampling after day seven are not indicated because short-term (acute) and long-term (latent) survivorship were evaluated separately.


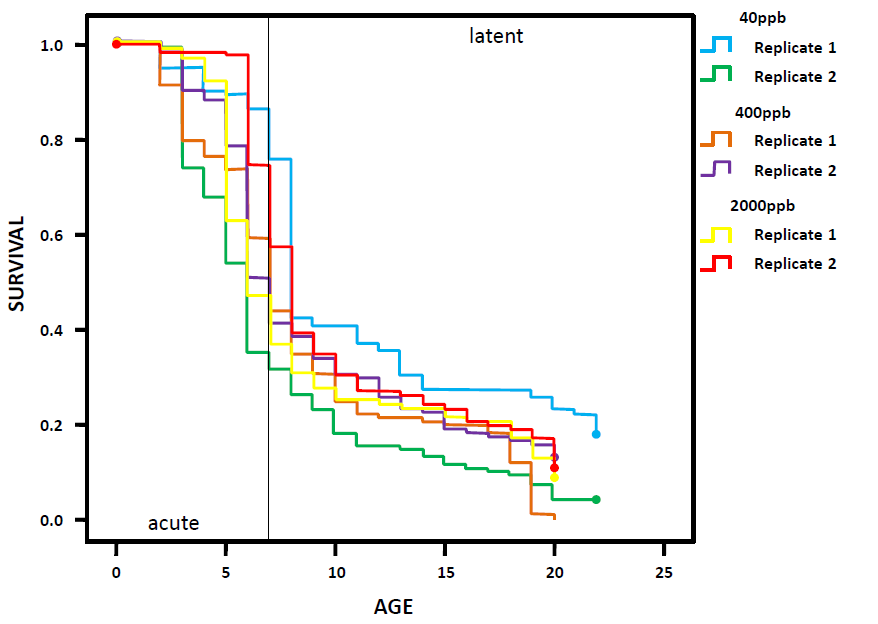


Figure S4: Cumulative survivorship of worker honey bees in the acetone control group. Censored data due to sampling after day seven are not indicated because short-term (acute) and long-term (latent) survivorship were evaluated separately.


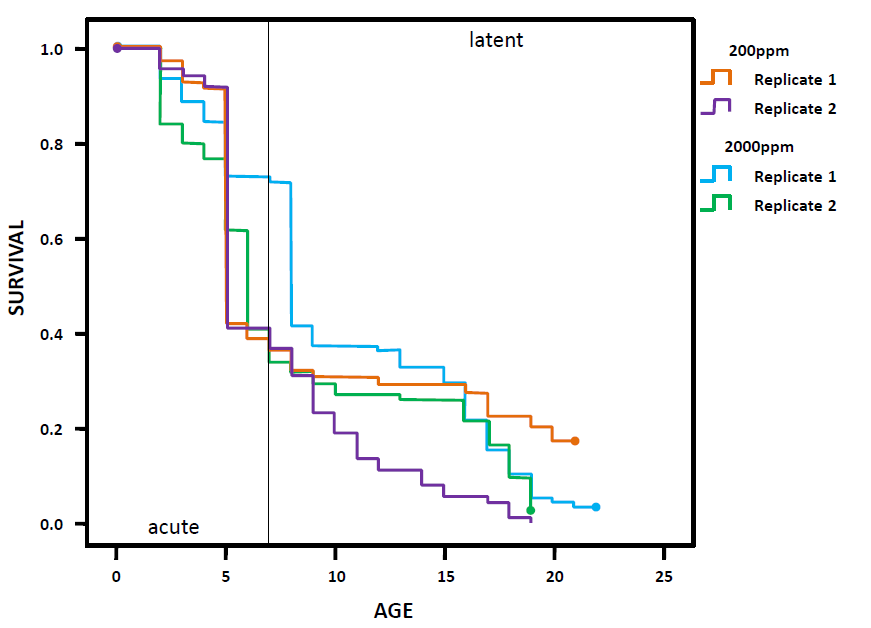


Figure S5: Cumulative survivorship of worker honey bees in the fluvalinate group. Censored data due to sampling after day seven are not indicated because short-term (acute) and long-term (latent) survivorship were evaluated separately.


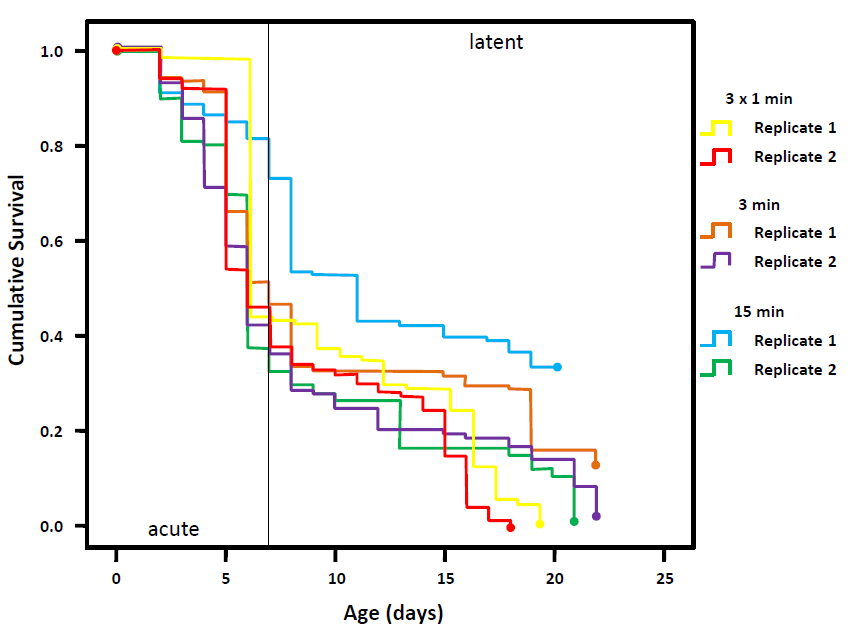


Figure S6: Cumulative survivorship of worker honey bees in the fluvalinate and coumaphos combination group. Censored data due to sampling after day seven are not indicated because short-term (acute) and long-term (latent) survivorship were evaluated separately.


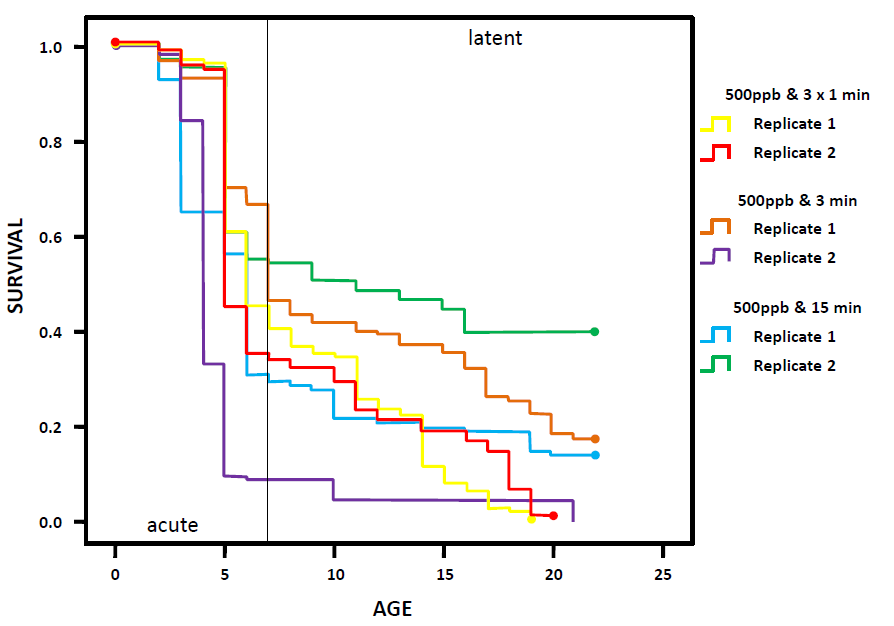

Supplement: File S1 — Significant variability in mortality among separate cages was observed within each treatment of the second experiment. This file details the mortality results with respect to the separate cages in each treatment. Due to unexplainable variation and the focus of our study on ISC proliferation, we omitted these details from the main text. (DOCX) [file pone.0091180.s001.docx]
